# Lung Ultrasonography Beyond the Diagnosis of Pediatrics Pneumonia

**DOI:** 10.7759/cureus.22460

**Published:** 2022-02-21

**Authors:** Adil Elabbas, Rabia Choudhary, Dedeepya Gullapalli, Shreeya Mistry, Farzana M.H, Abbas H Mallick, Eseoghene P Kevu, Javaria Asif, Jihan A Mostafa

**Affiliations:** 1 Radiology, California Institute of Behavioral Neurosciences & Psychology, Fairfield, USA; 2 Internal Medicine, California Institute of Behavioral Neurosciences & Psychology, Fairfield, USA; 3 Research, California Institute of Behavioral Neurosciences & Psychology, Fairfield, USA; 4 Psychiatry, California Institute of Behavioral Neurosciences & Psychology, Fairfield, USA

**Keywords:** imaging, cap, ultrasongraphy, pediatric penumonia, lung ultrasound (lus)

## Abstract

Pneumonia is a prevalent disease with considerable morbidity and mortality among the pediatric population. Early diagnosis and swift commencement of the correct treatment are vital for a favorable clinical outcome. Along with history-taking and clinical examination, imaging modalities commonly used, lung ultrasound provides a bedside, less invasive, radiation-free alternative to diagnose pneumonia when compared with other images such as chest x-ray (CXR) and computed tomography (CT) scan. It is therefore of the utmost magnitude to inspect the evidence of its accuracy and reliability in the diagnosis of this condition. The goal of this study is to look into the available data supporting the use of lung ultrasound in the diagnosis of juvenile pneumonia, its relevance in distinguishing between viral and bacterial diseases, and its superiority as compared to other diagnostic methods. As mentioned, early detection and differentiation of the type of pneumonia can reduce unnecessary antibiotic prescriptions and provide patients with a better prognosis, as well as the ability to predict the course of the disease and the need for advanced care or the development of complications. An extensive literature search of two popular online medical websites (PubMed and Embase) was conducted in this review, concentrating on studies that examined the role of lung ultrasound in the diagnosis of pediatric pneumonia published in the last five years. Only studies published in the English language were included in this review. With high sensitivity and specificity, lung ultrasound appeared to be a promising tool not only for pediatric pneumonia diagnosis, but also for treatment guidance and disease follow-up, especially when combined with clinical presentation and laboratory findings.

## Introduction and background

Chest infection is one of the leading causes of death among the pediatric population all around the world. According to the World Health Organization (WHO) data, pneumonia was responsible for the deaths of 808,694 children under the age of five, representing 15% of the overall mortality of the under-five group in 2017 [1,2]. Despite the fact that chest x-rays (CXRs) are widely used, routine use of chest radiography is not recommended due to a lack of spot diagnostic signs and symptoms and the radiation dosage [3-5]. Lung ultrasound (LUS) is now viewed as one of the preferable first-line modality tests for the diagnosis of pediatric pneumonia compared to conventional chest radiography [6,7]. A recent meta-analysis reported an overall high sensitivity (95.0%) and specificity (96.1%) of lung ultrasound in the diagnosis of pneumonia in children, as seen in Heuvelings et al. 2019’s paper [8]. As stated earlier, LUS is preferred to chest x-ray due to its high diagnostic accuracy (higher sensitivity and specificity). Not to mention that LUS is portable, quicker to administer, less expensive, simple to learn, and can be used at the bedside [9].

The purpose of this article is to review the available evidence for the utilization of lung ultrasound in the diagnosis of pneumonia in children and to show its role in differentiating between viral and bacterial pediatric pneumonia. Early detection and differentiation of the type of pneumonia (bacterial vs. viral) can reduce unnecessary antibiotic prescriptions and carry an overall better prognosis for patients, as well as the ability to predict the course of the disease and the need for advanced care or complication development.

## Review

Discussion

Pneumonia is a common and widespread disease in the pediatric population worldwide. Early diagnosis of pneumonia is vital for a favorable clinical outcome and, therefore, studying the accuracy of different methods of detecting the disease has become essential. 

A literature search of popular online medical websites (PubMed and Embase) was conducted for this current review, focusing on studies that examined the role of lung ultrasound in the diagnosis of pediatric pneumonia published in the last five years. Only studies published in the English language were included in this review. Performing a chest x-ray or a computed tomography (CT-scan) on a child comes with inherent side effects, mainly exposure to harmful radiation, which can even contribute to the development of malignancies. Besides the lack of diagnostic accuracy in CXR, low sensitivity and specificity, high cost, and time-consuming [10]. The obvious less invasive option to diagnose pneumonia has to be lung ultrasonography, as it does not have the above-mentioned risks [11]. It is of utmost importance, therefore, to examine the evidence of its accuracy and reliability in the diagnosis of this condition. Four systematic reviews and meta-analysis published recently have shown a range of sensitivity of lung ultrasound for the diagnosis of children's pneumonia from (93% to 95%) and a specificity ranging from (90% to 96.1%). The collective sensitivity and specificity of lung ultrasound in the diagnosis of pneumonia is summarized in Table 1.

**Table 1:** Summary of the collective sensitivity and specificity of lung ultrasound in the diagnosis of pediatric pneumonia.

The review study	Number of studies included	Total number of patients	Sensitivity	Specificity
Xin et al. [11]	8 Studies	1,013 Patients	93.0%	96.0%
Najgrodzka et al. [12]	19 Studies	3,361 Patients	93.16%	92.48%
Heuvelings et al. [8]	18 Studies	2,073 Patients	95.0%	96.1%
Yan et al. [7]	22 Studies	2,470 Patients	95%	90%

Approach of lung ultrasound in children

In order to obtain the best image resolution, a linear probe that gives a high frequency of 6 to 12 MHz is required [12,13]. At the start, the line of pleura is easily observed due to its location [14]. The lung ultrasound findings are viewed as artifacts, and the results are conveyed as the presence or absence of these artifacts. Careful interpretation and understanding of these artifacts are essential to reach the specific diagnosis [15-17].

Lung scanning can be carried out in various positions, which makes it very convenient for examining children of different age groups, ranging from newborns to adolescents. Optimal patient position for scanning is crucial to obtain the best possible images, which can be easily obtained in small babies. The test can be performed in the parent’s lap or even while breastfeeding to have easy compliance and to keep the neonate serene and calm. Probe disinfection before and after the procedure and heated lubricant can also be used to avoid stressing the child [17]. One of the main advantages of LUS, especially when compared to chest x-ray or CT-scan, is that it is not time-consuming. In a recent systematic review by Heuvelings et al., they reported a mean time of 7.4 minutes to complete the procedure [8]. Both sides of the chest are further divided into three areas: anterior, lateral, and posterior scanning of all the territory with different positioning [6]. When scanning the lung, a series of horizontal artifacts represent the sliding movement of the lungs during normal respiration. While the vertical artifact appears when the sonographic waves encounter the alveolar gas-liquid interface, lung consolidation is evident when the picture of the lung looks like a liver ultrasound scan, so-called "hepatization of the lung" [18]. Table 2 summarizes the most common ultrasound findings in pneumonia [18,19].

**Table 2:** Summary and description of the common lung ultrasound findings. US: ultrasound.

Ultrasound finding	Clinical significance	Description
A-line	Normal	Horizontal artifacts
B-lines	Pathological	Vertical artifacts
Lung consolidation	Pathological	Lung surface mimic liver US appearance
Pleural effusions	Pathological	Fluids in plural space

Lung consolidation, as depicted in the classic image, produces a sign known as the "shred sign." The shred sign is also known as the "alveolar syndrome." It indicates that the deep limit of the lungs seen on ultrasound is irregular when compared to normal lung ultrasound findings. This finding, however, is linked to a variety of pathologies, one of which is pneumonia [17].

**Figure 1:**
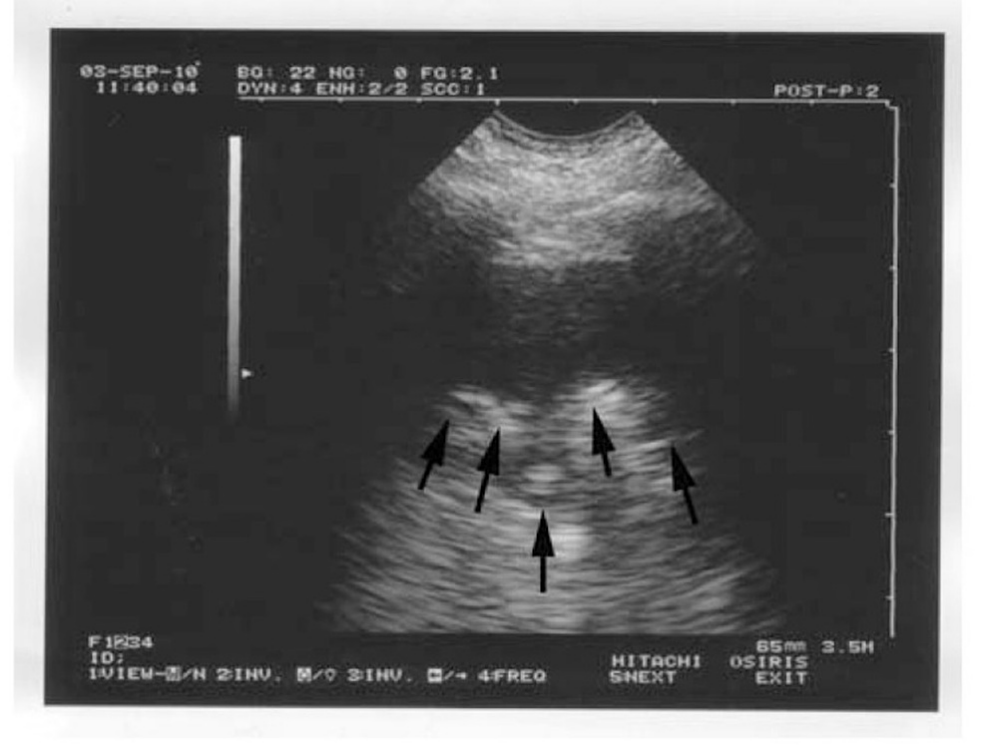
Arrows represent the shred sign, which is indicative of lung consolidation as seen in pneumonia. Adapted by Lichtenstein [17]. Copyright © 2012 Bentham Science Publishers. This is an open access article distributed under the terms of the Creative Commons Attribution License (http://creativecommons.org/licenses/by/2.5/), which permits unrestrictive use, distribution, and reproduction in any medium, provided the original work is properly cited.

Figure 1 demonstrates the radiological illustration of lung consolidation as seen in Lichtenstein's work [17]. The ultrasound of the lungs reveals that the deep limit (arrows) is irregular. The shredded sign is unique to lung consolidation and can be seen in pneumonia [17].

B lines are associated with extravascular lung water and may indicate interstitial or alveolar fluid. They can be seen in a variety of diseases, including pneumonia. This sign is extremely important in acute lung ultrasound in critically ill patients. The B-line is a comet-tail artifact that arises from the pleural line, is hyperechoic like the pleural line, spreads out without fading to the screen's edge, is well-defined, erases the A-lines, and moves in tandem with lung sliding. Lung rockets are three or more B-lines that are equivalent to the interstitial syndrome. They are used to distinguish between different types of acute respiratory failure and to aid in the management of acute circulatory failure [17].

**Figure 2:**
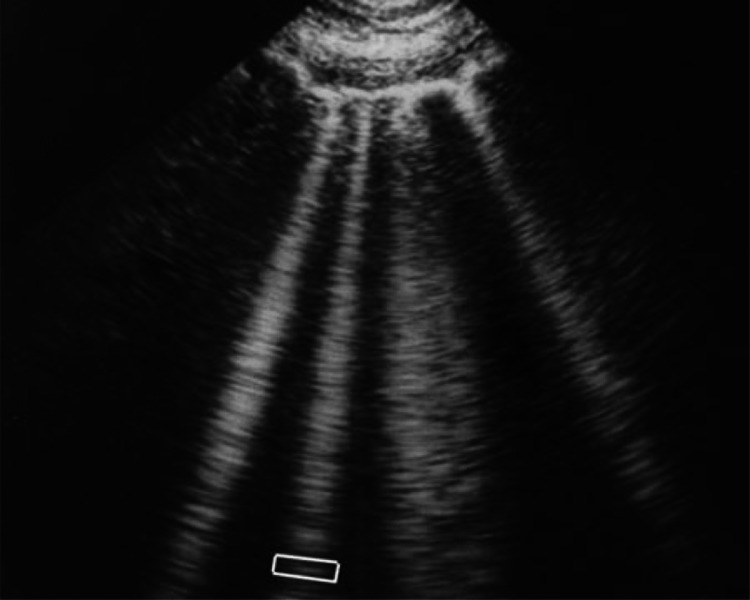
B-line is a vertical line shaped by numerous small horizontal lines, as seen in numerous pathologies, including bacterial or viral pneumonia. Adapted by Lichtenstein [17]. Copyright © 2012 Bentham Science Publishers. This is an open access article distributed under the terms of the Creative Commons Attribution License (http://creativecommons.org/licenses/by/2.5/), which permits unrestrictive use, distribution, and reproduction in any medium, provided the original work is properly cited.

Figure 2 shows vertical b lines that can be seen on lung ultrasound when diagnosing pneumonia, regardless of etiology, taken from Lichtenstein's paper [17].

Do we need to give antibiotics to all chest infections?

The similarity of all chest infection presentations, as well as the uncertainty surrounding the diagnosis, has contributed to the overuse of antibiotics in viral diseases [20,21]. The most commonly reported findings in pediatric pneumonia lung US are lung consolidation, the presence of an air bronchogram, abnormal changes in the pleural line, and fluid accumulation in the pleural space [22]. Recognizing the causative organism of the illness gives a simple answer about the targeted treatment, avoiding the unnecessary administration of antibiotics, reducing the health care burden, and decreasing the chance of developing more antibiotic resistance. LUS is deemed to have an important role in distinguishing between bacterial and viral chest infections in children and can facilitate the initiation of proper management [15]. Berce et al. demonstrated the ability of lung US to identify the causative organism of pediatric pneumonia [22]. Certain features, like having multiple consolidations, go with viral or atypical bacterial pneumonia compared to bacterial disease, which is usually presented as an isolated consolidation.

For the purpose of investigating the role of lung ultrasound in the diagnosis of community-acquired pneumonia (CAP) and examining its use in establishing the etiology of pneumonia, Berce et al. recruited a total of 147 children who were admitted to the hospital with CAP [22]. Initially, they included 180 hospitalized children (between the ages of one month and 16 years), but for various reasons, 41 subjects were excluded. Lung ultrasound was performed at admission, and the diagnosis of CAP was established using the British Thoracic Society (BTS) criteria. They reported the optimal cut-off point to differentiate between viral and bacterial pneumonia is a consolidation size of 21 mm for the diagnosis of bacterial CAP, with a sensitivity and specificity of 80% and 75%, respectively. Furthermore, they reported that lung ultrasound can differentiate between typical and atypical bacterial CAP. They cited a consolidation size of 21 mm to diagnose the former, with a sensitivity of 80% and specificity of 60%. However, lung ultrasound had no useful role in differentiating between viral and atypical bacterial CAP, as the findings on the ultrasound were somewhat similar for both [22].

The authors went on to conclude that the findings of lung ultrasound when examining patients with CAP are best interpreted in combination with epidemiological, clinical, and laboratory findings. However, clinicians should take these conclusions with a pinch of salt as they had somewhat strict inclusion and exclusion criteria. This means that the results cannot be generalized or applied to all children with CAP. For instance, children with immune deficiency, chronic lung disease, heart disease, neurological impairment, or any other condition that makes the children more likely to develop CAP were excluded. Moreover, only patients requiring hospital admission were included in the study, but those admitted to the intensive care unit (ICU) were excluded. In addition, patients who took a course of oral antibiotics prior to hospitalization were not excluded, even though they could interfere with ultrasound and laboratory findings. The authors have addressed some of these limitations and acknowledged that the differentiation between viral and bacterial pneumonia in children is arbitrary due to the lack of a gold-standard test [22].

Advancement in technology has made lung ultrasound an attractive option to consider for the diagnosis of pneumonia. To evaluate the usefulness of lung ultrasound in distinguishing between viral and bacterial pneumonia, Malla and colleagues conducted a prospective, cross-sectional study where they recruited a total of 200 children under the age of 12 with suspected pneumonia [15]. They had rather similar exclusion criteria to the previous study (Berce et al.), as they excluded hemodynamically unstable patients, those with pre-existing pulmonary disease, congenital heart defects, or those with pulmonary edema [15,22]. The authors noted that lung ultrasound was able to detect small consolidations. Additionally, and in agreement with Brece et al., they reported that a finding of a subpleural consolidation larger than 20 mm is indicative of bacterial pneumonia, whereas a subpleural consolidation between 5 and 20 mm is likely to be suggestive of viral pneumonia [15,22]. They reported that lung ultrasound has a high sensitivity (91%) and specificity (91.3%) in detecting bacterial pneumonia in children under the age of 12. However, lung ultrasound showed less sensitivity (78.4%) and specificity (90.4%) for diagnosing viral pneumonia [15]. They finally concluded that lung ultrasound has high precision in discriminating between bacterial and viral pneumonia in children and can help to reduce the unnecessary prescription of antibiotics. The study highlighted the issue of antibiotic over-prescription and reported that 77% of study subjects received antibiotics, whereas only 52.4% of children actually had bacterial pneumonia. Because the two studies had the same objective and had similar inclusion and exclusion criteria, it was not surprising to find that they shared similar limitations. One of the major limitations of studies trying to diagnose and distinguish between bacterial and viral causes of the disease is the lack of a gold-standard diagnostic test. In this study, the authors chose to use a combination of clinical picture, laboratory results, and chest radiograph as the gold standard for diagnosing pneumonia. Using a different reference standard is probably the reason why Malla et al. had a higher sensitivity and specificity compared to Berce et al. who used the BTS guidelines to diagnose pneumonia. Moreover, including chest radiographs as part of the gold standard is probably one of the biggest limitations of the Malla study [15,22]. They seem to be aware of that too, as they reported that small consolidations are unlikely to be identified by a chest x-ray. This was also reported by Shah et al. [23], who stated that lung consolidations measuring 10 mm or less are undetectable by chest x-ray. As the study was conducted on hospitalized patients, its findings cannot be generalized to include children who seek treatment in the outpatient setting. The authors mentioned that antibiotics were overly prescribed in their study subjects. However, they did not seem to note that receiving a course of antibiotics for days prior to hospital presentation could have interfered with findings on chest x-ray and ultrasound. The authors concluded that lung ultrasound is capable of diagnosing pediatric pneumonia based on the causative organism [15].

The importance lung ultrasound and follow-up for children with pneumonia

Musolino et al. 2019 work focused on the advantage of a repeatable radiation-free test with immediate bedside result and cost-effective. The lung US is considered as a useful tool for follow-up of pediatric pneumonia, treatment, and early detection of complications [24]. To examine the benefit of ultrasound in predicting the outcome of pneumonia in hospitalized children, Chen et al. conducted a retrospective study in which they reviewed the clinical records of 142 patients between the ages of six months and 18 months who were hospitalized between January 2010 and December 2012 [25]. All of these patients had trans-thoracic ultrasound within two days of admission. The authors described an association between some sonographic findings and the course of pneumonia in children. They found out that the presence of multifocal involvement (p value 0.0001) and pleural effusion (p value 0.001) in ultrasound were both associated with ICU admission. They also reported the following factors increasing the duration of hospital admission: the presence of multifocal involvement, increased B-lines, pleural effusion, and the presence of fluid in the bronchogram. The authors concluded that trans-thoracic ultrasound can play an important role not just in the diagnosis of pneumonia but also in predicting the course of the disease and outcome in hospitalized children [25].

A major limitation to the study, and one that they acknowledged, is the retrospective design of the study and the relatively small sample size. It is recommended that a prospective study with a larger sample size is needed to confirm these findings [25]. So, as mentioned earlier, LUS has a big role to play in the diagnosis of CAP, but can it do more than that? Is there a role for LUS in monitoring the response to antibiotic medications? To find out about that, Buonsenso et al. studied six children diagnosed with CAP, examining their clinical data, blood test results, and lung ultrasound findings [26]. In their case series, which was published in 2019, they described the changes in LUS findings after 48 hours of the commencement of antibiotic treatment. The first case they cited was a three-year-old girl who presented to the emergency department with symptoms and blood investigations consistent with CAP. At presentation, the main LUS finding was a large area of consolidation. Two days after the commencement of intravenous antibiotics, LUS was repeated, and it showed more aerated lung fields and smaller consolidation. She was discharged, and a follow-up ultrasound showed normal findings. The second case was that of a 13-month-old infant who also presented to the ED with a fever and a lack of appetite. The clinical picture and laboratory findings were consistent with CAP as well. On the day of admission to the pediatric ward, a lung ultrasound was performed, and it showed a large consolidation and a branching superficial air bronchogram. After two to 48 hours of treatment, the LUS showed a smaller consolidation and a more aerated lung field. Despite the small sample size the author, went on to conclude that the ultrasound changes following treatment with antibiotics in children with CAP were pretty consistent. Changes in lung consolidation size and bronchogram distribution were more closely related to treatment response or failure than blood tests or clinical pictures [26]. Finally, Buonsenso et al. went on to say that LUS seems like a promising tool for the personalized management of CAP. Of course, caution should always be exercised when interpreting the results and recommendations of case series and case studies as oftentimes they are influenced by the author’s experience and personal opinion, and there is no control of confounding factors. The extent to which the results of case series can be generalized is also limited, and they are placed at the bottom of the hierarchy of evidence [26].

Musolino et al. were also interested in finding out if LUS could be used to monitor treatment outcomes in children diagnosed with CAP. They went for a prospective study design, and they performed LUS at baseline (T0) and after 48 hours of antibiotic treatment, aiming to investigate the reliability of LUS in the prediction of complicated CAP too. Their study participants, a total of 101, included patients aged from one month to 17 years. The authors reported that all of their patients had subpleural parenchymal lesions greater than one centimeter. They also reported that a bigger lesion was strongly associated with a longer hospitalization. Despite repeating LUS only after 48 hours, it was noticed that those parenchymal lesions had completely disappeared in 26.7% of study participants, which is in line with findings of previous studies [24]. For example, Lanniello et al. reported that after five days of treatment, LUS showed the complete disappearance of parenchymal lesions or at least a decrease in their size; this reportedly happened in 86.4% of their patients [27]. Similar to what Buonsenso et al. reported in their case series, an air bronchogram was one of the most common LUS findings in children with CAP [26]. However, Musolio et al. further reported that children with complicated CAP and longer hospitalization were more likely to show a deep, fixed air and liquid bronchogram compared to those with uncomplicated CAP. According to the authors [24], this difference was statistically significant.

The author then concluded that LUS was able to describe the early changes that happen in the lungs secondary to CAP and that it can also be helpful in predicting those who will develop complicated CAP. They finally added that LUS can be useful for clinicians in the management of children with CAP. They did acknowledge some of the study limitations and named selection bias as one of the major limitations, as patient participants were chosen according to the clinical need for imaging. Bias does limit the conclusions and recommendations that could be drawn from the analysis. Selection bias occurs when there are systematic differences between those who were chosen to participate and those who were left out. Even though they wished to study LUS changes in complicated CAP and how they differ from uncomplicated cases, they based this comparison on a small group of participants only. Of course, that does not make the findings of this study invalid; it just means that one has to bear these limitations in mind when thinking about implementing recommendations from this study.

Pitfalls of lung ultrasound

Despite the relative ease of learning, ultrasound remains dependent on the operator's technique, with expert operators producing the most accurate images. Unlike chest x-ray, lung ultrasound doesn't provide full visualization of the lung field. One of the limitations of lung ultrasound is the difficulty of observing some anatomical areas, namely, the area underneath the bony structures such as the scapula and clavicle. Visualization of the perihilar areas can also be challenging when using ultrasound. More importantly, tiny and localized parenchymal lesions do not reach the pleura, making them hard to detect by ultrasound [26-28].

Limitations

The majority of the articles in this review were based on hospitalized patients or critically ill cases, and it should be noted that the study population was small, with no gold-standard test for the diagnosis of pediatric pneumonia. Studies used a variety of references, ranging from clinical diagnosis to CT and MRI. The reference images are frequently reported by experienced radiologists compared to the bedside ultrasound images, which were analyzed by the attending clinician. The diversity of experience of the sonographic examiner represents different results. Some suggest the type of ultrasound machine used and the quality of images could be another factor [29].

## Conclusions

In conclusion, pediatric pneumonia is a common cause of death in children, and its diagnosis and management are of eminent importance. Along with the clinical evaluation and laboratory results, it can be diagnosed by imaging studies such as chest x-ray, lung ultrasound, and CT scan, which carry high radiation risks. Ultrasound showed high sensitivity and specificity without radiation risk. The goal of this study was to assess the available evidence supporting the use of lung ultrasound in the diagnosis of juvenile pneumonia, its relevance in distinguishing between viral and bacterial pneumonia, and its superiority as compared to other diagnostic methods. Early detection and differentiation of the type of pneumonia can reduce unnecessary antibiotic prescriptions and provide patients with a better prognosis, as well as the ability to predict the course of the disease and the need for advanced care or the development of complications. 

When combined with clinical presentation and laboratory findings, lung ultrasound appears to be a promising tool not only in the diagnosis of pediatric pneumonia but also in treatment guidance and follow-up of the disease. However, further studies with larger sample size and a pragmatic design are needed. In addition, studies looking into community cases of pediatric pneumonia are lacking as well.
